# Event-triggered state estimator design for unknown input and noise-correlated random system

**DOI:** 10.1016/j.heliyon.2020.e03832

**Published:** 2020-05-16

**Authors:** Liu He, Yingjun Zhao, Qingkuan Dong

**Affiliations:** a Air and Missile Defense College, Air Force Engineering University, Xi'an 710051, China; b State Key Laboratory of Integrated Services Networks (ISN), Xidian University, Xi'an 710071, China

**Keywords:** Electrical engineering, Computer science, Computer security and privacy, Network (Computer science), Ad Hoc network, Computer network, Internet, Network security, Wireless sensor network, Event-triggered, State estimator, Unknown input, Correlated noises

## Abstract

In this study, an event-driven state estimator is designed for stochastic systems that contain unknown inputs and processes as well as correlated measurement noise. First, the event-triggered state estimator's gain is deduced by using the random stability theory and Lyapunov's function. Then, based on the results, the corresponding state estimation errors are calculated in mean square convergence. Second, the corresponding unknown inputs are inhibited by using output errors of the estimator. In addition, the corresponding event-driven transmission strategy is designed by using a quadratic performance index, which guarantees a good balance between the estimation error and the data transmission rate as well as prolonged service life of the sensor battery. Finally, numerical simulation tests verify that the designed event-driven state estimator can estimate the system's state effectively and extend the sensor's battery life by approximately 48%. The proposed algorithm also leads to reduced utilization of network resources to some degree.

## 1 Introduction

Wireless sensor networks have been widely used in areas such as intelligent transportation systems, environmental monitoring, and industrial automation [1]. Replacement of old batteries incurs high cost and new batteries are difficult to procure due to limited availability. To solve these problems, an approach of reducing the rate of communication between sensors and estimators, and thus the battery energy consumption, has been proposed [2]. Although this approach cannot guarantee perfect estimation performance, it provides a possibility for event-triggered transmission schemes to maintain a balance between the estimation error and the communication rate between sensors and estimators. State estimation of periodic transmission [3, 4] at equal intervals requires large network bandwidth. Therefore, it is of great importance to a construct event-driven sensor data transmission mechanism that determines when a sensor packet needs to be sent to the remote end. DAWEI et al. [5] considered the problem of event-triggered state estimation for linear time-invariant systems under a maximum likelihood framework. WU et al. [6] analyzed an online sensor transmission strategy with timeout. However, these studies ignore the correlation between process noise and measurement noise. In addition, unknown inputs such as unknown disturbance, faults, and modeling uncertainties are not considered. Therefore, in this study, the state estimation of stochastic systems based on event-driven transmission considering unknown inputs and processes is investigated, and an event-driven estimator for stochastic systems with unknown inputs and processes and measurement noise is proposed. The stability of the designed estimator is proved by using the stochastic Lyapunov theory, and the corresponding estimator gain is obtained by using the linear matrix inequality (LMI) approach [[7], [8], [9]]. Based on the performance index of a quadratic approximate system, the corresponding event-driven transmission strategy is proposed, which aims to strike a balance between the estimation error and the communication rate between sensors and estimators [[10], [11]].

This paper presents an event-driven control strategy that considers unknown inputs and correlated measurement noise for detecting the anomalous events caused by them. For achieving this objective, the state estimator gain triggered by events is derived by a stochastic Lyapunov function using stochastic stability theory. The corresponding mean square error of state estimation is compensated and the unknown inputs and correlated noise are suppressed using the output error of the estimator. Further, the event-driven transmission strategy is designed on the basis of approximate secondary performance indicators.

## 2 Problem statement

**Figure 1:**
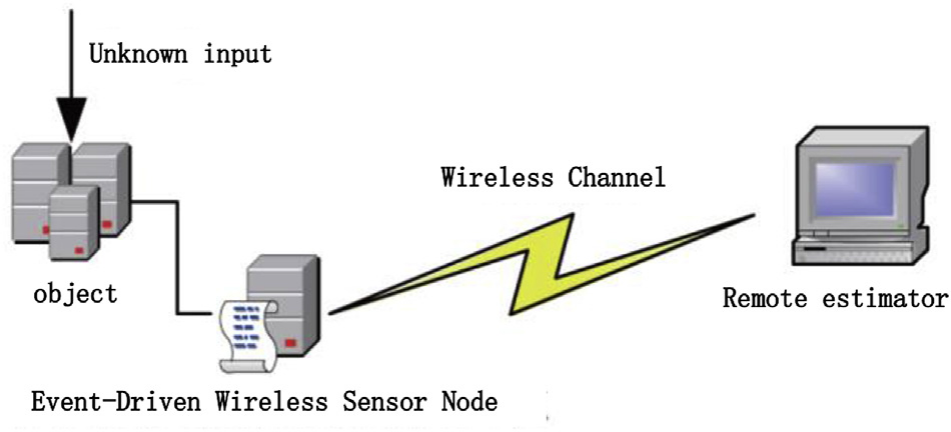
The event-triggered state estimator driving the plan of sensor.

State estimation based on the measurement process is achieved with a battery-powered sensor, as shown in Figure 1, when an event is triggered, the current sensor measurements are sent to the remote estimator. Because of the correlation between process noise and measurement noise, the standard Kalman filter cannot be applied. In this study, we will relax the assumption of the standard Kalman filter as $E\left[w_{k}v_{j}^{T}\right]\neq 0\forall k,j$, where E[⋅] represents the mathematical expectation operator and T refers to the matrix transposition.

The process dynamics and sensor measurement equations are assumed to be as follows:

(1) $$x_{k+1}=Ax_{k}+w_{k}+d_{k}$$

(2) $$y_{k}=Cx_{k}+v_{k}$$

In the equation, $x_{k}\in R^{n}$ is the state of the system, ${\text{R}}^{m\;\times \;n}$ represents the real matrix of m×n, and $y_{k}\in R^{m}$ is the measured value of the sensor at time k. $d_{k}\in R^{n}$ is an unknown input or disturbance, and noise sequences $w_{k}$ and $v_{k}$ are zero-mean Gaussian random noise with covariance.

(3) $$E\left[v_{k}v_{j}^{T}\right]=Q_{w}\delta_{kj}\geq 0$$

(4) $$E\left[v_{k}v_{j}^{T}\right]=\text{R}\delta_{kj}>0$$

(5) $$\;E\left[w_{k}v_{j}^{T}\right]=S\delta_{k-j+1}>0\forall k,\;j$$

From Eqs. (1), (2), (3), (4), and (5), we have found the dependency between k and k+1, and come to the conclusion that the correlation between $w_{k}$ and $v_{j}$ is formed by physical process since both of them are external disturbance inputs [12]. The initial state $x_{0}$ is also a zero-mean Gaussian random vector that is not related to $w_{k}$ and $v_{k}$, and its covariance $P_{0}\geq 0$. We assume that (C,A) is observable and $\left(A,\sqrt{Q_{w}}\right)$ is controllable, then unknown input $d_{k}$ does not affect noise $\left\{w_{k}\right\}$ and $\left\{v_{k}\right\}$. When $y_{k}$ needs to be transmitted to the remote estimator, the event-driven algorithm determines whether to send it to the estimator. Consider $\gamma_{k}$ as the decision variable: $\gamma_{k}=1$ means $y_{k}$is sent, and $\gamma_{k}=0$ means that it is not sent. Therefore, the true value of $y_{k}$ is known only when $\gamma_{k}=1$. Next, we present the optimal event-driven recursive estimation as follows:

(6) $${\hat{x}}_{k+1}=\left\{ \begin{array}{l} A{\hat{x}}_{k}+\bar{K}\left(y_{k}-C{\hat{x}}_{k}\right)\;\gamma_{k}=1\\ A{\hat{x}}_{k}\;\;\;\;\;\;\;\;\;\;\;\;\gamma_{k}=0 \end{array}\right.$$

And the state error $e_{k}$ satisfies the following equation:

(7) $$\begin{array}{l} e_{k}=x_{k}-{\hat{x}}_{k}=\\ \left(A-\gamma_{k-1}\bar{K}\mathrm{CA}\right)e_{k-1}-\gamma_{k-1}\bar{K}v_{k}+\\ \left(I-\gamma_{k-1}\bar{K}C\right)w_{k-1}+d_{k-1} \end{array}$$

**Lemma 1** (Lemma 1 [13]): Define $V\left(e_{k}\right)$ as a Lyapunov function. If there exist $\varepsilon_{1}\geq 0$, $\varepsilon_{2}>0$, $\varepsilon_{3}>0$ and $0<\varepsilon_{4}\leq 1$, then

(8) $$\varepsilon_{2}{\left\|e_{k}\right\|}^{2}\leq V\left(e_{k}\right)\leq \varepsilon_{3}{\left\|e_{k}\right\|}^{2}$$

and

(9) $$E\left\{V\left(e_{k+1}|e_{k}\right)\right\}-V\left(e_{k}\right)\leq \varepsilon_{1}-\varepsilon_{4}V\left(e_{k}\right)$$

Then the mean $e_{k}$ square is bounded. As shown in

(10) $$E\left\{{\left\|e_{k}\right\|}^{2}\right\}\leq \frac{\varepsilon_{3}}{\varepsilon_{2}}{\left\|e_{0}\right\|}^{2}{\left(1-\varepsilon_{4}\right)}^{k}+\frac{\varepsilon_{1}}{\varepsilon_{2}\varepsilon_{4}}\sum_{i=1}^{k}{\left(1-\varepsilon_{4}\right)}^{k}$$

Next, defining T∈N as the time domain, and selecting J as a cost function, we get

(11) $$J=\underset{T\to \infty }{\lim\;sup}\frac{1}{T}\sum_{k=0}^{T-1}E\left(b\left(e_{k}\right)\right)$$

the $b\left(e_{k}\right)=e_{k}^{T}He_{k}+\theta \gamma_{k}$, the system weight H>0 and the communication weight θ>0.

The cost given by Eq. (11) is called the approximate quadratic performance index [14]. Design in accordance with the secondary form defined by *H* and the number of data transmissions set as $\gamma_{k}=1$. The main tool used to determine the upper bound of the performance index is given by the following lemma.

**Lemma 2** (Theorem 1 [15]): Suppose there is a Markov sequence that satisfies the state space X. Suppose that:f:X→R, b:X→R. Definition:

(12) $$J=\underset{T\to \infty }{\lim\;sup}\frac{1}{T}\sum_{k=0}^{T-1}E\left(b\left(x_{k}\right)\right)$$

If there is c∈R which satisfies

(13) m(x)≥cx∈X,

then it can be concluded that

(14) $$J\leq \underset{\vartheta \in X}{\sup}\left(b\left(\vartheta \right)+E\left(m\left(x_{k+1}\right)|x_{k}+\vartheta \right)-m\left(\vartheta \right)\right)$$

## 3 Design of event-driven estimator

Here, the $\gamma_{k}=1$ controller gain K is derived by Lemma 1 to achieve convergence of the mean square of state $x_{k}$ and suppress the effect of $d_{k}$.

***Theorem 1*:** If $\gamma_{k}=1$ is given a positive number $\gamma_{1}$, P>0 is assumed to be a symmetric matrix, and the following linear inequality is satisfied

(15) $$\left[\begin{array}{cccc} -P & A^{T}P-A^{T}C^{T}{\bar{L}}^{T} & C^{T} & A^{T}P-A^{T}C^{T}{\bar{L}}^{T}\\ PA-\bar{L}CA & P-\gamma_{1}^{2}I & 0 & 0\\ C & 0 & -I & 0\\ PA-\bar{L}CA & 0 & 0 & -P \end{array}\right]<0$$

Then, the mean square of state $e_{k}$ is bounded when $d_{k}=0$. Further, when $d_{k}\neq 0$, under the initial condition of 0, the output $\left\|y_{k}-C{\hat{x}}_{k}\right\|\leq \gamma_{1}\left\|d_{k}\right\|$. The controller gain $\bar{K}=P^{-1}\bar{L}$.

**Proof:** To satisfy the condition (8) of Lemma 1, we select the following Lyapunov function

$$V\left(e_{k}\right)=e_{k}^{T}Pe_{k},$$

P>0 is a symmetric matrix. When $d_{k}=0$, $w_{k}\neq 0$ and $v_{k}\neq 0$, by substituting formula (7) into the above formula

$$\begin{array}{l} E\left\{\left(V_{k+1}|e_{k},...,e_{0}\right)\right\}-V\left(e_{k}\right)=e_{k}^{T}\Psi^{T}P\Psi e_{k}+\\ trace\left({\bar{K}}^{T}P\bar{K}R\right)-trace\left(\tilde{S}\right)+trace\left({\hat{B}}^{T}\hat{P}BQ_{w}\right)-e_{k}^{T}Pe_{k} \end{array}$$

and

$\Psi =A-\bar{K}CA,\;\hat{B}=I-\bar{K}C,\;\tilde{S}=S^{T}{\hat{B}}^{T}P\bar{K}+{\bar{K}}^{T}P\hat{B}S,$ $\bar{K}=P^{-1}\bar{L}$, in order to satisfy the condition of Lemma 9, if $\Psi^{T}P\Psi -P<0$, the above formula can be simplified as

$$\begin{array}{l} E\left\{\left(V_{k+1}|e_{k},...,e_{0}\right)\right\}-V\left(e_{k}\right)<-\beta e_{k}^{T}e_{k}+\\ trace\left({\bar{K}}^{T}P\bar{K}R\right)+trace\left({\hat{B}}^{T}\hat{P}BQ_{w}\right) \end{array}$$

here $\Omega_{11}=\Psi^{T}P\Psi -P,\;0<\beta <\min\left\{\lambda_{\min}\left(-\Omega_{11}\right),\lambda_{\max}\left(P\right)\right\},$$\lambda_{\min}\left(\cdot \right)$ and $\lambda_{\max}\left(\cdot \right)$ refer to minimum and maximum eigenvalues of the matrix, respectively.

By using Lemma 1 and Inequality (15), the above formula can be summarized as

$$E\left\{{\left\|e_{k}\right\|}^{2}\right\}\leq \frac{\lambda_{\max}\left(P\right)}{\lambda_{\min}\left(P\right)}{\left\|e_{0}\right\|}^{2}{\left(1-\beta \right)}^{k}+\frac{trace\left({\bar{K}}^{T}P\bar{K}R\right)+trace\left({\hat{B}}^{T}\hat{P}BQ_{w}\right)}{\lambda_{\min}\left(P\right)\beta }$$

Therefore, it is verified that when $d_{k}=0$ and $\gamma_{k}=1$, $e_{k}$ is mean-square bounded.

Next, assuming $d_{k}\neq 0$ , $w_{k}=v_{k}=0$, we reformulate $\Delta V_{k}$ as

$$\begin{array}{l} \Delta V_{k}=E\left\{\left(V_{k+1}|e_{k},...,e_{0}\right)\right\}-V\left(e_{k}\right)=e_{k}^{T}\Psi^{T}P\Psi e_{k}+\\ trace\left({\bar{K}}^{T}P\bar{K}R\right)-trace\left(\tilde{S}\right)+trace\left({\hat{B}}^{T}\hat{P}BQ_{w}\right)-\\ e_{k}^{T}Pe_{k}+d_{k}^{T}Pd_{k}+2e_{k}^{T}\Psi^{T}Pd_{k} \end{array}$$

Introducing performance index $H_{\infty }$ to suppress the effect of unknown inputs $d_{k}$, we get

$$J_{1}=E\left\{\sum_{k=0}^{\infty }{\left(y_{k}-C{\hat{x}}_{k}\right)}^{T}\left(y_{k}-C{\hat{x}}_{k}\right)\right\}-\gamma_{1}^{2}E\left\{\sum_{k=0}^{\infty }d_{k}^{T}d_{k}\right\}$$

Under the initial conditions of 0, $J_{1}$ is substituted in $\Delta V_{k}$, as

$$J_{1}\leq E\left\{\sum_{k=0}^{\infty }\left(\left(e_{k}^{T}\;d_{k}^{T}\right)\left[\begin{array}{ll} \Omega_{11}+C^{T}C & \Psi^{T}P\\ P\Psi & P-\gamma_{1}^{2}I \end{array}\right]\left(\begin{array}{l} e_{k}\\ d_{k} \end{array}\right)\right)\right\}$$

If $\Omega =\left[\begin{array}{ll} \Omega_{11}+C^{T}C & \Psi^{T}P\\ P\Psi & P-\gamma_{1}^{2}I \end{array}\right]<0$, then the following inequality can be obtained

$$E\left\{\sum_{k=0}^{\infty }{\left(y_{k}-C{\hat{x}}_{k}\right)}^{T}\left(y_{k}-C{\hat{x}}_{k}\right)\right\}-\gamma_{1}^{2}E\left\{\sum_{k=0}^{\infty }d_{k}^{T}d_{k}\right\}\leq 0$$

Therefore, we can conclude the following

$$\left\|y_{k}-C{\hat{x}}_{k}\right\|\leq \gamma_{1}\left\|d_{k}\right\|$$

With Schur complement, we can get the following matrix inequality constraints

(26) $$\left[\begin{array}{cccc} -P & A^{T}P-A^{T}C^{T}{\bar{L}}^{T} & C^{T} & A^{T}P-A^{T}C^{T}{\bar{L}}^{T}\\ PA-\bar{L}CA & P-\gamma_{1}^{2}I & 0 & 0\\ C & 0 & -I & 0\\ PA-\bar{L}CA & 0 & 0 & -P \end{array}\right]<0$$

The $\bar{L}=P\bar{K}$.

The proof is completed.

## 4 Design of event-driven strategy

In this section, we derive the event-driven transmission strategy by using Lemma 2 to prove the upper bound of the approximate quadratic performance.

Theorem 2: Suppose M>0 is a symmetric matrix, θ>0 as communication weight. If:

(16) $$\begin{array}{l} H-M+\Psi^{T}M\Psi +\hat{\theta }\sum \leq 0,\\ ATMA-M+H-\lambda \sum \leq 0,\;\hat{\theta }\geq 0 \end{array}$$

and

(17) $$\begin{array}{l} \hat{\theta }=\theta -trace\left(\tilde{S}\right)+trace\left({\hat{B}}^{T}\hat{P}BQ_{w}\right)+\\ \;\;trace\left({\bar{K}}^{T}P\bar{K}R\right)-\lambda \end{array}$$

(18) $$\sum =C^{T}\frac{\theta }{traceH}C,\;\hat{R}=\frac{\theta }{traceH}R$$

(19) $$\Psi =A-\bar{K}CA,\;\hat{B}=I-\bar{K}C,\;\hat{S}=S^{T}{\hat{B}}^{T}M\bar{K}+{\bar{K}}^{T}M\hat{B}S$$

The event-driven transport strategy is:

$${\left(y_{k}-C{\hat{x}}_{k}\right)}^{T}-\frac{\theta }{trace\left(H\right)}\left(y_{k}-C{\hat{x}}_{k}\right)\leq trace\left(\hat{R}\right),\;\text{then}\;\gamma_{k}=0,\;\text{otherwise}\;\gamma_{k}=1.$$

The approximate quadratic performance function satisfies

(20) $$J\leq \lambda +trace\left(Q_{w}M\right)$$

**Proof:** choose a function $m\left(e\right)=e^{T}Me$. It is easy to see that m(e) is an upper bound, $m\left(e_{k+1}\right)$ can be calculated by (7)

$$e_{k+1}=\left\{ \begin{array}{l} Ae_{k}+w_{k}\gamma_{k}=0\;\;\;\;\\ \Psi e_{k}-\bar{K}v_{k+1}+\hat{B}w_{k}\gamma_{k}=1 \end{array}\right.$$

Then the following formula can be obtained as

$$\begin{array}{l} E\left(m\left(e_{k+1}\right)|e_{k}=\vartheta \right)=\\ \left\{ \begin{array}{l} \vartheta^{T}A^{T}MA\vartheta +trace\left(MQ_{w}\right)\;\;\;\gamma_{k}=0\\ \vartheta^{T}\Psi^{T}M\Psi \vartheta +trace\left({\bar{K}}^{T}M\bar{K}R\right)-\;\;\\ trace\left(\hat{S}\right)+trace\left({\hat{B}}^{T}\hat{M}BQ_{w}\right)\;\gamma_{k}=1 \end{array}\right. \end{array}$$

Define the function *g*:

$$g\left(\vartheta \right)=b\left(\vartheta \right)+E\left(m\left(e_{k+1}\right)|e_{k}=\vartheta \right)-m\left(\vartheta \right),$$

Substituting the above formula, we get:

$$\begin{array}{l} g\left(\vartheta \right)=\\ \left\{ \begin{array}{l} \vartheta^{T}A^{T}MA\vartheta +trace\left(MQ_{w}\right)-\vartheta^{T}M\vartheta +\vartheta^{T}H\vartheta \;\;\;\gamma_{k}=0,\\ \vartheta^{T}\Psi^{T}M\Psi \vartheta -\vartheta^{T}M\vartheta +\vartheta^{T}H\vartheta +\theta -trace\left(\hat{S}\right)+\;\;\;\\ trace\left({\hat{B}}^{T}\hat{M}BQ_{w}\right)+trace\left({\bar{K}}^{T}M\bar{K}R\right)\;\;\;\;\;\;\;\;\gamma_{k}=1 \end{array}\right. \end{array}$$

In order to derive Lemma 2, we need to calculate the upper bound. First, when $\gamma_{k}=1$, we get

$${\left(y_{k}-C{\hat{x}}_{k}\right)}^{T}-\frac{\theta }{trace\left(H\right)}\left(y_{k}-C{\hat{x}}_{k}\right)>trace\left(\hat{R}\right)$$

We get

$$\begin{array}{l} g(\vartheta )-trace(MQ_{w})=\vartheta^{T}(\Psi^{T}M\Psi -\\ \;\;\;\;\;\;\;\;M+H)\vartheta +\hat{\theta }-trace(\hat{S}) \end{array}$$

Considering $H-M+\Psi^{T}M\Psi +\hat{\theta }\sum \leq 0$

We get

$$\begin{array}{l} (\theta -trace(\hat{S})+trace({\hat{B}}^{T}\hat{M}BQ_{w})+trace({\bar{K}}^{T}M\bar{K}R)-\\ \lambda )\vartheta^{T}\sum \vartheta \leq \vartheta^{T}(M-\Psi^{T}M\Psi -H)\vartheta \end{array}$$

Because

$${\left(y_{k}-C{\hat{x}}_{k}\right)}^{T}-\frac{\theta }{trace\left(H\right)}\left(y_{k}-C{\hat{x}}_{k}\right)>trace\left(\hat{R}\right)$$

We can use expectations to give:

$$\begin{array}{l} E\left({\left(y_{k}-C{\hat{x}}_{k}\right)}^{T}-\frac{\theta }{trace\left(H\right)}\left(y_{k}-C{\hat{x}}_{k}\right)\right)>\\ \;\;\;trace\left(\hat{R}\right),\;\vartheta^{T}\sum \vartheta >1. \end{array}$$

Since $\hat{\theta }\geq 0$, we get

$$\begin{array}{l} \theta -trace\left(\hat{S}\right)+trace\left({\hat{B}}^{T}\hat{M}BQ_{w}\right)+\\ trace\left({\bar{K}}^{T}M\bar{K}R\right)-\lambda \leq \vartheta^{T}\left(M-\Psi^{T}M\Psi -H\right)\vartheta \end{array}$$

Therefore, we can be inferred that

$$g\left(\vartheta \right)\leq \lambda +trace\left(MQ_{w}\right).$$

then

$${\left(y_{k}-C{\hat{x}}_{k}\right)}^{T}-\frac{\theta }{trace\left(H\right)}\left(y_{k}-C{\hat{x}}_{k}\right)\leq trace\left(\hat{R}\right)$$

we get

$$g\left(\vartheta \right)-trace\left(MQ_{w}\right)=\vartheta^{T}\left(A^{T}MA-M+H\right)\vartheta$$

then $A^{T}MA-M+H-\lambda \sum \leq 0$ and λ>0

we get

$$\vartheta^{T}\left(A^{T}MA-M+H\right)\vartheta \leq \lambda \vartheta^{T}\sum \vartheta$$

whereby ${\left(y_{k}-C{\hat{x}}_{k}\right)}^{T}-\frac{\theta }{trace\left(H\right)}\left(y_{k}-C{\hat{x}}_{k}\right)>trace\left(\hat{R}\right)$ we can infer

$$\begin{array}{l} E\left({\left(y_{k}-C{\hat{x}}_{k}\right)}^{T}-\frac{\theta }{trace\left(H\right)}\left(y_{k}-C{\hat{x}}_{k}\right)\right)\leq \\ \;\;\;trace\left(\hat{R}\right),\;\vartheta^{T}\sum \vartheta \leq 1. \end{array}$$

becomes $g\left(\vartheta \right)\leq \lambda +trace\left(MQ_{w}\right).$

If the assumption (16) can be established, the following conclusions can be drawn

$$J\leq \underset{\vartheta \in R^{n}}{\sup}g\left(\vartheta \right)\leq \lambda +trace\left(MQ_{w}\right).$$

The proof is completed.

## 5 Simulation

To prolong the battery life of wireless sensor networks, this study investigates through simulation the event-triggered state estimation of linear systems with intermittent measurements, referring to [16, 17].

We considered a process with a state space model:

$$\begin{array}{l} x_{k+1}=\left[\begin{array}{ll} 0.6 & 0\\ 1 & 0.8 \end{array}\right]x_{k}+w_{k}+d_{k},\\ y_{k}=\left[\begin{array}{ll} 1 & 0\\ 0 & 1 \end{array}\right]x_{k}+v_{k}, \end{array}$$

which

$$\begin{array}{l} Q_{w}=\left[\begin{array}{ll} 0.2 & 0.043\\ 0.043 & 0.3 \end{array}\right],\;S=\left[\begin{array}{ll} 0.25 & 0\\ 0 & 0.25 \end{array}\right],\\ R=\left[\begin{array}{ll} 1 & 0\\ 0 & 1 \end{array}\right],\;d=\left[\begin{array}{l} \sin\left(x_{1,k}+x_{2,k}\right)\\ \sin\left(x_{1,k}+x_{2,k}\right) \end{array}\right] \end{array}$$

In addition, $\gamma_{2}=0.2$, furthermore, the error weighted value, the transmission weighted value and the event-driven gain are calculated by Theorem 2, where we get

$$H=\left[\begin{array}{ll} 3 & 2\\ 2 & 1 \end{array}\right],\;\lambda =11,\;\bar{K}\approx \left[\begin{array}{ll} 0.8761 & 0\\ 0 & 0.8340 \end{array}\right]$$

In order to simplify the simulation complexity, the average approximate quadratic performance index is given in this paper:

$$J_{avg}=\frac{1}{T}\sum_{k=0}^{T-1}\left({\left(x_{k}-{\hat{x}}_{k}\right)}^{T}H\left(x_{k}-{\hat{x}}_{k}\right)+\lambda \gamma_{k}\right)$$

The system simulations were implemented in a MATLAB environment. Simulation of periodical control under each scenario was performed 50 times starting in the initial zero state, according to the standard procedures.

**Figure 2:**
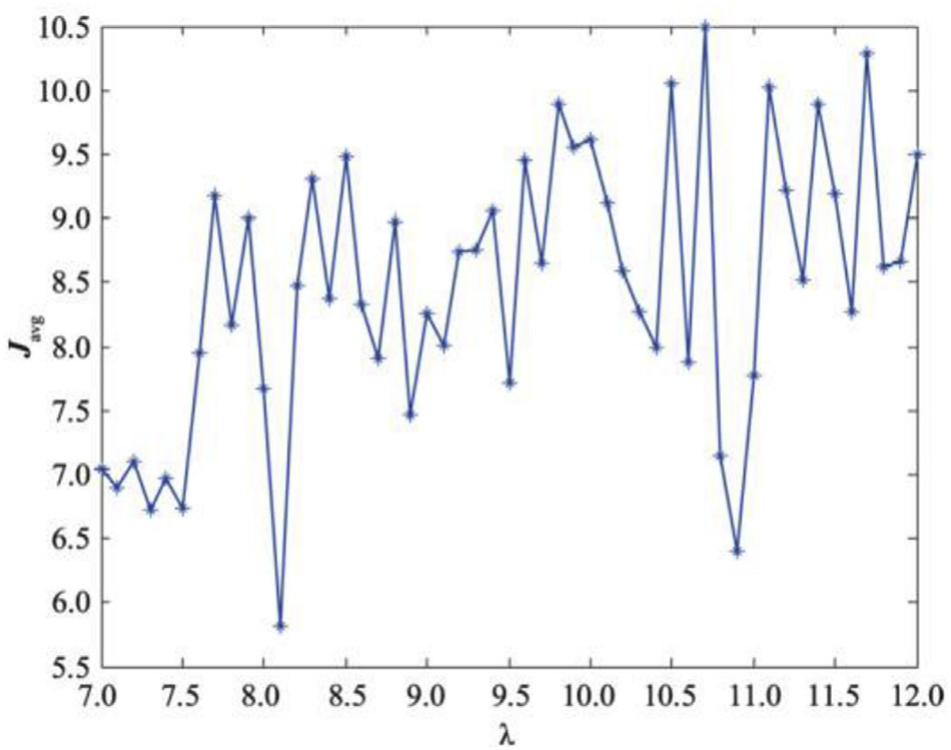
The limit of the $J_{\text{avg}}$.

**Figure 3:**
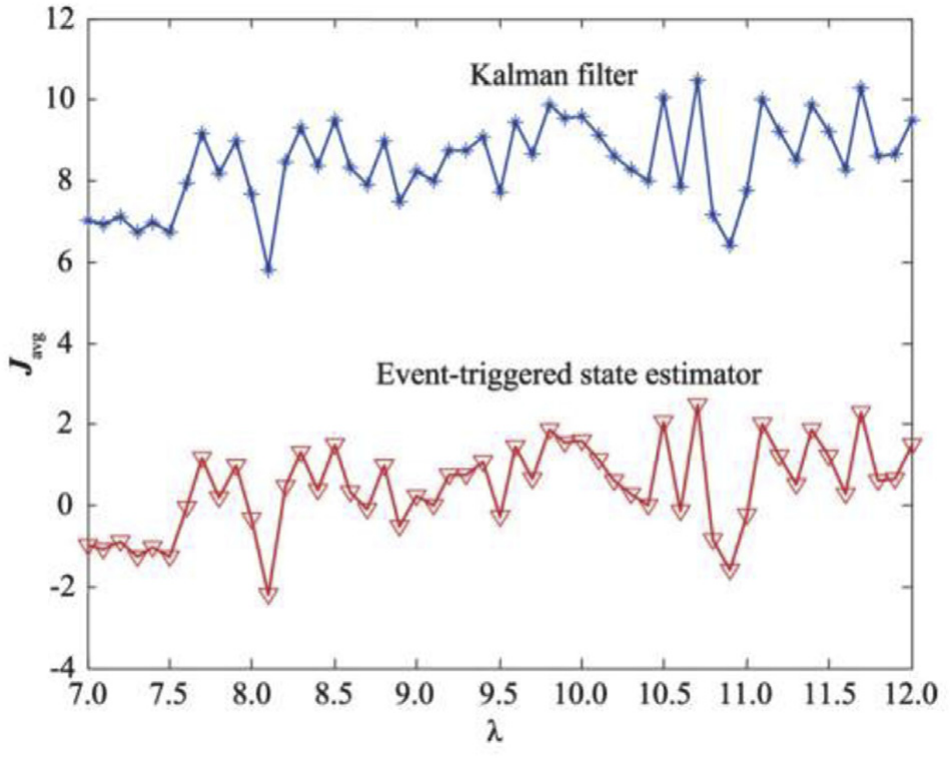
Compare with the $J_{\text{avg}}$ of Kalman filter.

Under this performance indicator, the upper performance limit of the estimator with varying transmission weight λ is shown in Figure 2. Figure 3 shows the comparison of the results of the proposed estimator and the standard Kalman estimator. Both results are consistent, which further proves that the proposed transmission strategy can balance the estimation performance and communication rate more effectively compared to periodic transmission.

To study the improvement of the battery life of wireless sensors, a first-order radio model was applied in this study. In this model, the radio consumes battery energy at a rate of $E_{elec}=50nJ•bit^{-1}$. By adjustingθ, the energy consumption estimated by the proposed method is plotted in 100 time steps.

**Figure 4:**
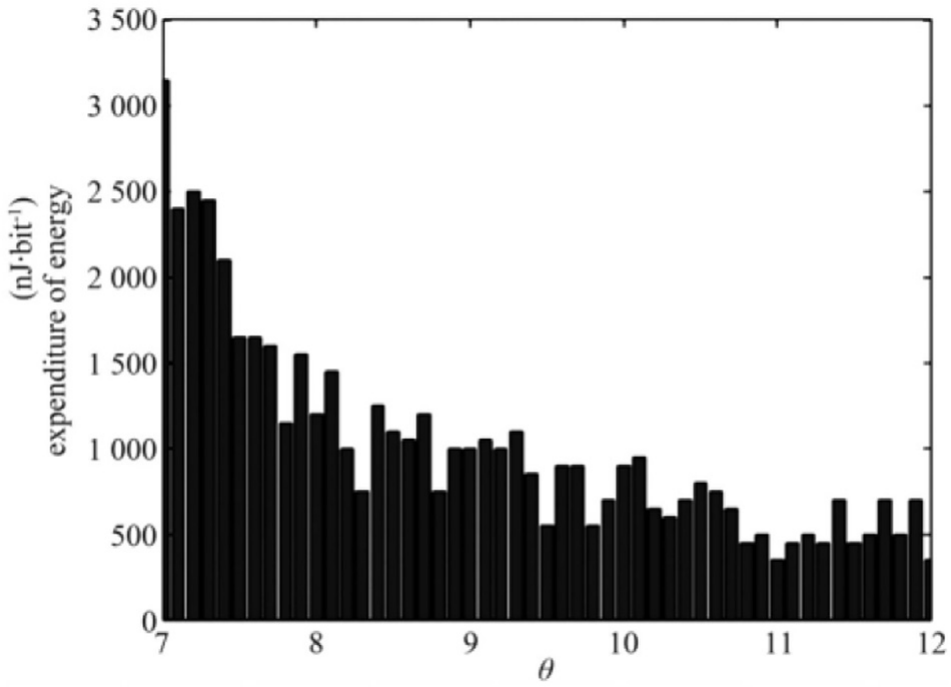
The energy consumption for sensor.

Figure 4 shows that when θ=7, the energy consumed by the battery is $E_{elec}\approx 2500nJ•bit^{-1}$, and it gradually reduces to $E_{elec}\approx 1200nJ•bit^{-1}$ as θ gradually increases. This phenomenon implies that by increasing θ, that is, reducing the number of data transmissions, the energy consumed by the power supply battery of the sensor can be gradually decreased, extending the battery life by approximately 48% (1200/2500 = 48%).

**Figure 5:**
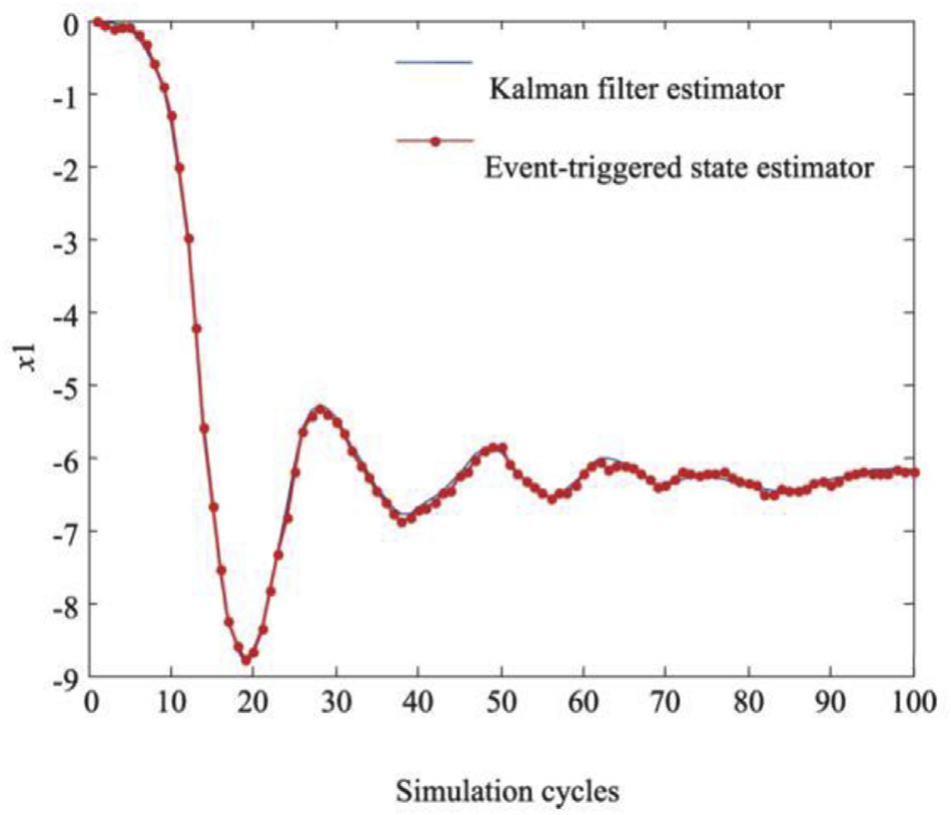
Tracking effect of status x1.

**Figure 6:**
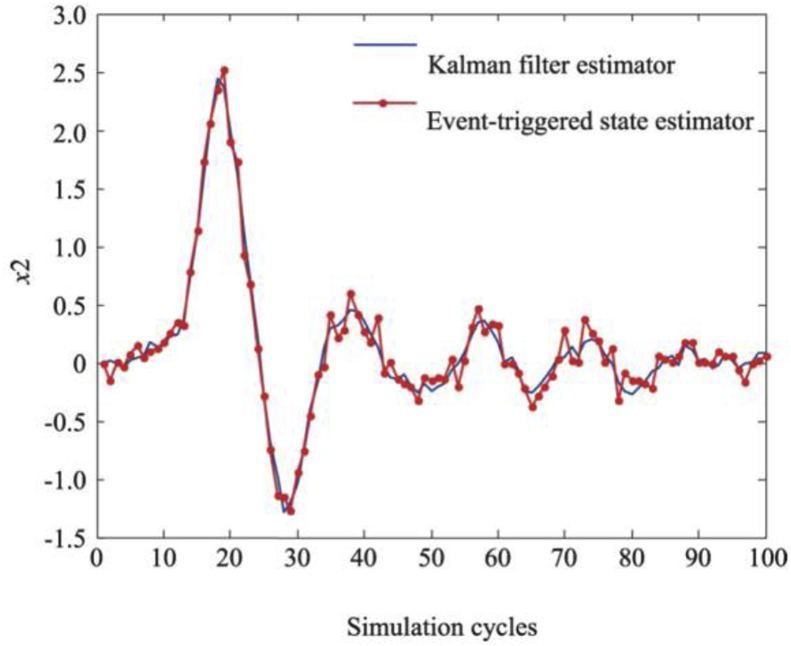
Tracking effect of status x2.

To check the performance of the designed estimator, the system was compared with the standard Kalman estimator by performing the simulation in the initial zero state 100 times in a MATLAB environment. Figures 5 and 6 show that the difference between the mechanisms of event-driven transmission and the periodic transmission is small; that is, the system performance is not greatly affected by using event-driven transmission. Although the use of an event-driven transmission mechanism reduces the transmission rate of measurements, the tracking effect is comparable to that of a time-driven standard Kalman estimator; this proves that the proposed algorithm can balance the estimation performance and the number of data transmissions between sensors and estimators well. Thus, the proposed event-driven state estimator achieves the purpose of prolonging battery life and reducing the requirement of the wireless communication bandwidth.

## 6 Conclusion

In this study, we proposed an event-driven state estimator for stochastic wireless sensor systems under unknown inputs and correlated noise. Unknown inputs and correlated noise affect the state at time k+1 in the state model; therefore, correlated noise is defined as the process correlated noise at time k and the measurement correlated noise at time k+1. First, using the results of the stochastic Lyapunov stability theory, the homotopic boundedness of the error dynamics equation was derived. Second, considering the case where unknown input and correlated noise are not zero and using the classic $H_{\infty }$ performance indicator, the effect of correlated noise was suppressed. Furthermore, the corresponding estimator gain was derived by linear matrix inequalities, thereby ensuring the convergence of the mean square error when there are no unknown inputs and correlated noise. By contrast, in the case of deceitful inputs, the performance indicators based on the mean square output error were used to suppress the effect of correlated noise. Subsequently, an event-driven sensor transmission mechanism was derived to determine when data transmission should occur from a sensor. To obtain the optimal balance between the number of communications and the estimation error, the upper bound of the secondary performance index was derived using the result of Lemma 2 to obtain the optimal threshold for the transmission mechanism. Finally, simulations showed that the estimator and event-driven transmission strategy designed in this study can reconstruct the state of the system robustly and extend the service life of the sensor battery by approximately 48%, which is of a great significance.

## Declarations

### Author contribution statement

Liu He: Conceived and designed the experiments; Performed the experiments; Analyzed and interpreted the data; Contributed reagents, materials, analysis tools or data; Wrote the paper.

Qingkuan Dong: Conceived and designed the experiments; Analyzed and interpreted the data; Wrote the paper.

Yingjun Zhao: Performed the experiments; Contributed reagents, materials, analysis tools or data; Wrote the paper.

### Funding statement

This research did not receive any specific grant from funding agencies in the public, commercial, or not-for-profit sectors.

### Competing interest statement

The authors declare no conflict of interest.

### Additional information

No additional information is available for this paper.
